# Cdo Regulates Surface Expression of Kir2.1 K^+^ Channel in Myoblast Differentiation

**DOI:** 10.1371/journal.pone.0158707

**Published:** 2016-07-05

**Authors:** Young-Eun Leem, Hyeon-Ju Jeong, Hyun-Ji Kim, Jewoo Koh, KyeongJin Kang, Gyu-Un Bae, Hana Cho, Jong-Sun Kang

**Affiliations:** 1 Department of Molecular Cell Biology, Sungkyunkwan University School of Medicine, Suwon, Republic of Korea; 2 Department of Physiology, Sungkyunkwan University School of Medicine, Suwon, Republic of Korea; 3 Department of Anatomy, Sungkyunkwan University School of Medicine, Suwon, Republic of Korea; 4 Samsung Biomedical Research Institute, Suwon, Republic of Korea; 5 Research Center for Cell Fate Control, Sookmyung Women’s University, Seoul, Republic of Korea; National University of Singapore, SINGAPORE

## Abstract

A potassium channel Kir2.1-associated membrane hyperpolarization is required for myogenic differentiation. However the molecular regulatory mechanisms modulating Kir2.1 channel activities in early stage of myogenesis are largely unknown. A cell surface protein, Cdo functions as a component of multiprotein cell surface complexes to promote myogenesis. In this study, we report that Cdo forms a complex with Kir2.1 during myogenic differentiation, and is required for the channel activity by enhancing the surface expression of Kir2.1 in the early stage of differentiation. The expression of a constitutively active form of the upstream kinase for p38MAPK, MKK6(EE) can restore Kir2.1 activities in Cdo-depleted C2C12 cells, while the treatment with a p38MAPK inhibitor, SB203580 exhibits a similar effect of Cdo depletion on Kir2.1 surface expression. Furthermore, *Cdo*^*-/-*^ primary myoblasts, which display a defective differentiation program, exhibit a defective Kir2.1 activity. Taken together, our results suggest that a promyogenic Cdo signaling is critical for Kir2.1 activities in the induction of myogenic differentiation.

## Introduction

Formation of skeletal muscle is coordinated by differentiation and fusion of mononucleated myoblasts into multinucleated myofibers [1]. For the efficient coordination of myogenesis, it is required to regulate the expressions and activities of the MyoD family of transcription factors including Myf5, MyoD, Myogenin and MRF [2,3,4]. Various extracellular cues, such as cell adhesion and signaling pathways regulate the expression and activation of these transcription factors which are tightly associated with the initiation and maintenance of myoblast differentiation. Cell-cell interaction between myogenic precursors is critical for induction of myoblast differentiation. Stimulation of cell adhesion receptor complexes induces various signaling pathways including p38MAPK, Akt and ERK/Stim-1/Ca^2+^ pathways leading to activation of MyoD, NFATc3 and downstream target genes in myoblast differentiation [5,6,7,8].

Cdo is a cell surface receptor of the Ig superfamily that is expressed in muscle precursor cells and developing muscles during mouse embryogenesis [9,10]. It has been known to positively regulate myogenic differentiation as a component of a multiprotein complex consisting of Boc, Gas1, N-Cadherin and Neogenin/Netrin-3 [11,12,13,14]. The functional interaction between Cdo and Cadherins particularly makes Cdo to be considered as a strong protein molecule to mediate some of cell-cell contact-mediated effects which are critical in myogenesis. Cdo deficiency caused defects in myoblast differentiation or skeletal muscle development in mice [7,10]. Whereas, the ectopic expression of Cdo promoted myogenic differentiation in C2C12 myoblasts [9]. Cdo mediates its promyogenic activity via activation of key myogenic kinases, Akt and p38MAPK which can phosphorylate MyoD or E-protein resulting in muscle-specific gene expression. In addition, Cdo is implicated in the calcium-mediated signaling pathways required for myoblast differentiation. Our previous studies have suggested that Netrin2/Cdo signaling induces activation of NFATc3 and Stim-1 which enhances myoblast differentiation [15]. The coordinated regulation of multiple signaling pathways and transcription regulators by Cdo multiprotein complexes appears to be critical for the efficient myoblast differentiation and maintenance of the differentiated states.

The function of Kir2.1 K^+^ channels in myoblast differentiation is well documented [16,17]. Previous studies have suggested that a membrane hyperpolarization mediated by Kir2.1 K^+^ channel is one of the events which are primarily required for human myoblast differentiation by modulating expression of myogenic transcription factors, such as Myogenin and MEF2. Inhibition of the membrane hyperpolarization has been associated with the reduction in expressions and activities of Myogenin and MEF2 [17]. The Kir2.1-mediated hyperpolarization activates Ca^2+^-dependent Calcineurin pathway [18] which activates NFATc3 to promote myogenic differentiation. Kir2.1 activities can be regulated at the multiple levels including gene expression, membrane trafficking and open states of the channel [19,20]. For the membrane targeting of Kir2.1, signaling pathways, such as Rac1 has been shown to regulate Kir2.1 trafficking [21]. In addition, Kir2.1 activities can be enhanced by the protein stabilization at the plasma membrane through interaction with anchoring proteins, such as filamin A [22], PSD93δ [23], or SAP97 [24]. The activation of the membrane-resident Kir2.1 is regulated by several other signaling pathways, including PIP_2_-binding [25,26], PKA [27], PKC [28,29], and receptor-activated tyrosine kinases [30,31,32,33].

As aforementioned, both Kir2.1 and Cdo are required for myoblast differentiation and promote differentiation via common regulatory mechanisms such as Calcineurin/NFATc3 and Myogenin induction. Thus, we investigated a potential crosstalk between Cdo and Kir2.1 channels in myoblast differentiation. Cdo-depleted or deficient myoblasts exhibited impaired differentiation and a decreased Kir2.1 channel activity, without significant changes in Kir2.1 protein levels. Kir2.1 and Cdo were effectively coprecipitated in C2C12 cells. Moreover, in the same cells, Kir2.1 membrane levels were increased for at least 8 hours after differentiation; Cdo depletion impaired such increase in Kir2.1 membrane-residence occurring during the early stages of differentiation. Furthermore, the inward rectifying K^+^ channel activities induced upon differentiation were sensitive to p38MAPK inhibition and it seemed to be due to Kir2.1 surface trafficking. These data suggest that Cdo-mediated signaling might be involved in regulation of Kir2.1 trafficking and activation during myogenic differentiation.

## Results

**Fig 1:**
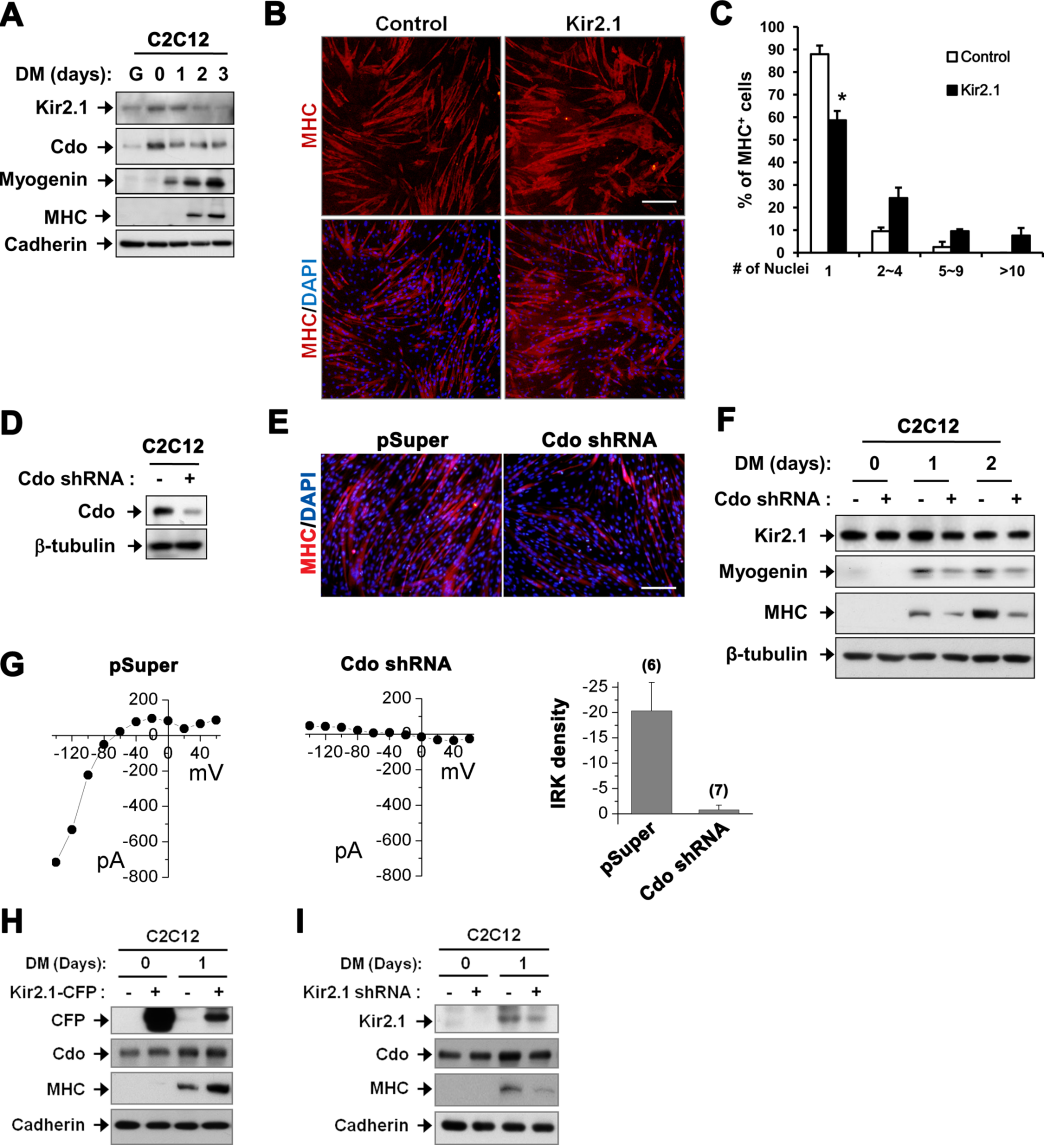
The reduction in Kir2.1 activity impaired myoblast differentiation in Cdo-depleted cells. (A) Western blot analysis of C2C12 cell lysates from various differentiation time courses for indicated proteins. (B) C2C12 cells were stably transfected with the control (pcDNA3.1) or Kir2.1 expression vectors, cultured in DM for 3 days and subjected to immunostaining with anti-MHC antibody (red). Cell nuclei were visualized by DAPI staining (blue). Scale bar indicates 200μm. (C) Quantification of the MHC-positive cells shown in B. Values represent means ± SEM in three random fields. *P <0.05. (D) Western blot analysis for expression of Cdo or β-tubulin in lysates of C2C12/pSuper and C2C12/Cdo shRNA cells, which were differentiated for 3 days. β-tubulin was used as a loading control. (E) C2C12 cells were stably transfected with pSuper or Cdo shRNA expression vectors. Cells were then induced to differentiate for 3 days and analyzed by immunostaining with anti-MHC antibody (red). Cell nuclei were visualized by DAPI staining (blue). Scale bar = 200μm. (F) Western blot analysis for expression of Kir2.1, Myogenin and MHC in C2C12/pSuper or C2C12/Cdo shRNA cells, which were induced to differentiate for indicated time courses. β-tubulin was used as a loading control. (G) Current-voltage relationships of inwardly rectifying K^+^ (IRK) current of C2C12 cells expressing either pSuper (left) or Cdo shRNA (middle). IRK current was obtained by subtraction of steady state currents at the end of test pulse, in the absence or presence of 0.5 mM Ba^2+^. Voltage-steps were from -140 mV to +60 mV from a holding potential at -70 mV. The graph on the right is the summary of IRK density. The current density is the whole-cell current amplitude divided by the membrane capacity (which is directly correlated with the surface area of the cell), thus allowing comparison between cells. IRK amplitude was measured during a step to –140 mV from a holding potential of –70 mV. (H) Western blot analysis for expression of CFP, Cdo and MHC in C2C12/Control or C2C12/Kir2.1-CFP cells, which were induced to differentiate for indicated time. Cadherin was used as a loading control [8,11,34]. (I) Western blot analysis for expression of Kir2.1, Cdo and MHC in C2C12/Control or C2C12/Kir2.1 shRNA cells, which were induced to differentiate for indicated time. Cadherin was used as a loading control.

To assess the crosstalk between Cdo and Kir2.1 channels, we have examined the expression pattern of these proteins in C2C12 myoblast differentiation. C2C12 cells were cultured at low cell density in growth medium with 15% fetal bovine serum (G), and at near-confluency (DM0), cells were induced to differentiate by culturing with the differentiation medium containing 2% horse serum (DM) for 3 days (DM3). Cdo and Kir2.1 were expressed at G and they were transiently increased upon differentiation (Fig 1A). According to the previous studies, the increase in Kir2.1 and Cdo proceeded Myogenin expression [9,16]. Similarly to the previous studies in human myoblasts, Kir2.1 overexpression in C2C12 myoblasts enhanced myoblast differentiation evident by the formation of larger myosin heavy chain (MHC)-positive myotubes with more nuclei per myotube (Fig 1B and 1C). Since both Cdo and Kir2.1 were induced upon myoblast differentiation, we assessed whether impaired myoblast differentiation caused by Cdo depletion was linked with a reduction in Kir2.1 expression. C2C12 cells were stably transfected with control (pSuper) or Cdo shRNA expression vectors and cells were subjected to DM. In agreement with the previous studies [9,14], Cdo-depleted C2C12 myoblasts that exhibited impaired myoblast differentiation displayed strongly reduced expression of Myogenin and MHC, compared to the control cells (Fig 1D–1F). However, the expression of Kir2.1 protein was not significantly altered in Cdo-depleted cells (Fig 1F). Since the channel activity can be regulated by changes in the surface trafficking or the open probability without altered expression levels, we then examined whether Kir2.1 activities were altered in Cdo-depleted C2C12 myoblasts by using a patch clamp whole-cell configuration. As shown in Fig 1G, Cdo-depleted cells showed significantly less average of Kir2.1 current density at –140 mV (-0.8±0.9 pA/pF, n = 7) compared with the control cells (-20.3±5.6 pA/pF, n = 6, *P* <0.01) after one day-exposure to DM. Then, we examined whether the enhanced myoblast differentiation by the ectopic expression of Kir2.1 is associated with the elevation of Cdo level. The result showed that Cdo expression was not influenced by the higher level of Kir2.1 (Fig 1H). Conversely, the depletion of Kir2.1 by shRNA did not alter the level of Cdo, although myogenic differentiation was hindered indicated by the reduction in MHC expression (Fig 1I). Taken together, these data suggest that the impaired myoblast differentiation caused by Cdo depletion is linked to the reduction in Kir2.1 activity.

**Fig 2:**
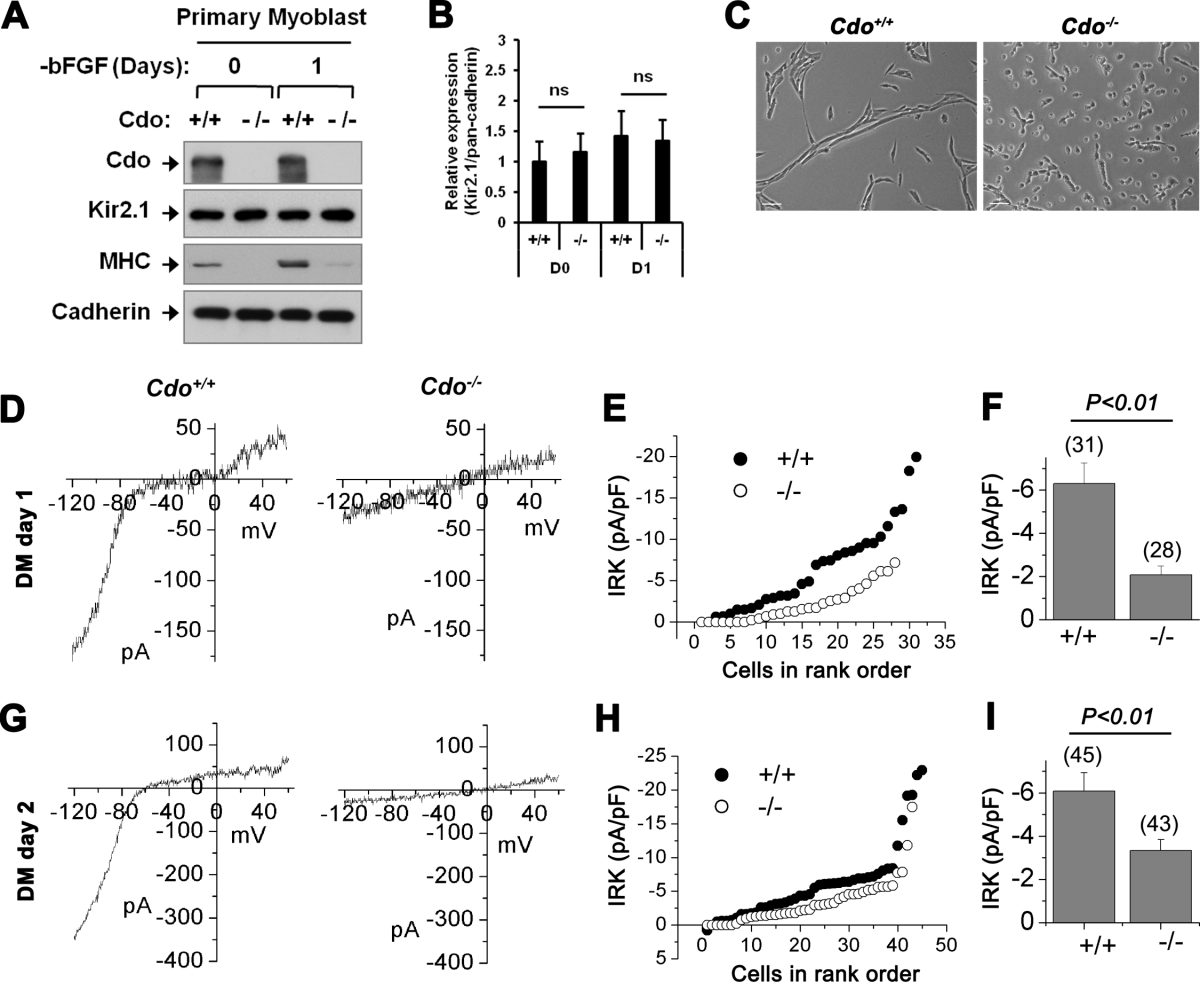
The myoblasts from *Cdo*^*-/-*^ mice exhibited the reduction in Kir2.1 activity and in myogenic differentiation. (A) Western blot analysis of the lysates of *Cdo*^*+/+*^ or *Cdo*^*-/-*^ myoblasts from various differentiation time points for indicated proteins. Cadherin was used as a loading control. (B) Quantification of the relative expression of Kir2.1, compared to the Cadherin levels shown in A. Values represent means ± SEM. (n = 3) ns, not significant. (C) Phase-contrast microscopic images of *Cdo*^*+/+*^ or *Cdo*^*-/-*^ myoblasts differentiated for 2 days. Size bar = 20μm. (D-I) IRK density was recorded in cells at differentiation day 1 (D-F) or 2 days (G-I). (D&G) Representative Ba^2+^-sensitive current trace of *Cdo*^*+/+*^ (left) and *Cdo*^*-/-*^ (right) primary myoblast kept in DM. *I*–*V* relationships were obtained from the current response to the voltage ramp from -120 mV to +60 mV. (E&H) Closed and open circles represent IRK density of *Cdo*^*+/+*^ and *Cdo*^*-/-*^ myoblast, respectively. IRK amplitude was measured in whole-cell configuration during a step to –140 mV lasting 1s from a holding potential of –70 mV. Each symbol represents one single cell. For each of *Cdo*^*+/+*^ and *Cdo*^*-/-*^ myoblast, cells were ordered from the smallest to the largest current density recorded. (F&I) Histograms of the average IRK density obtained from each population.

Next, we have further confirmed the effect of Cdo deficiency on Kir2.1 activities in primary mouse myoblasts. In consistent with the previous data (Fig 1F), Cdo deficiency had no effects on the Kir2.1 expression level, though it significantly impaired myoblast differentiation (Fig 2A–2C). We then examined whether *Cdo*^*-/-*^ myoblasts exhibited the reduction in Kir2.1 activities. At the differentiation day one, *Cdo*^*+/+*^ myoblasts exhibited large Kir2.1 currents, but *Cdo*^*-/-*^ myoblasts showed little Kir2.1 activities (Fig 2D and 2E). The current densities of wildtype and *Cdo*^*-/-*^ myoblasts at -120 mV were -6.3±0.9 pA/pF (*n* = 31) and -2.1±0.4 pA/pF (*n* = 28, *P*<0.01), respectively (Fig 2F). The reduction of Kir2.1 current densities in *Cdo*^*-/-*^ cells was maintained even after of 2 day-exposure to DM (Fig 2G–2I).

**Fig 3:**
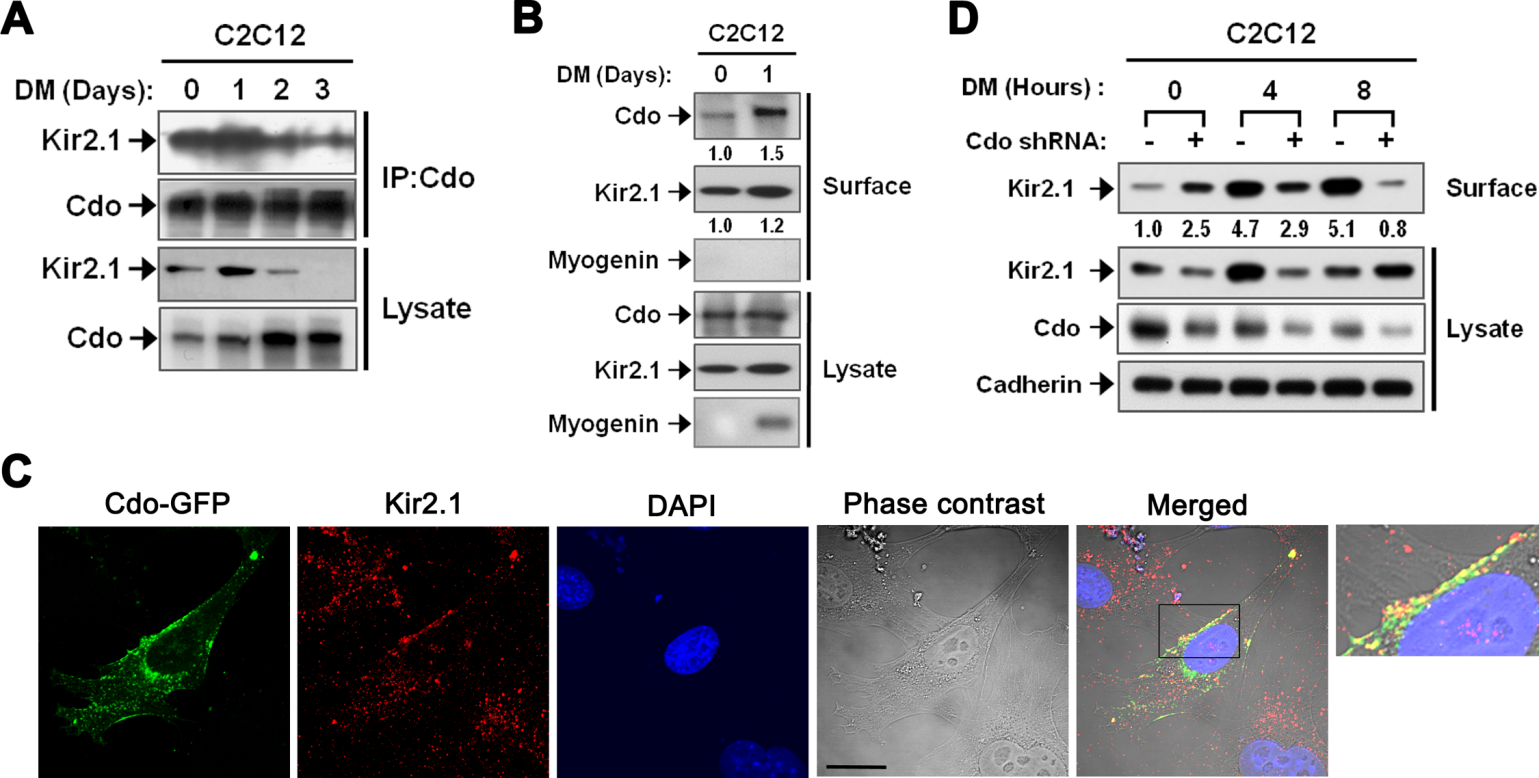
Cdo regulated Kir2.1 activity by modulating membrane localization of Kir2.1. (A) Co-immunoprecipitation of Kir2.1 with Cdo. Cell lysates were prepared from C2C12 cells differentiated for various time courses. Extracts were immuneprecipitated with an anti-Cdo antibody and blotted with an anti-Kir2.1 or anti-Cdo antibody. (B) Differentiated C2C12 cells for indicated time were subjected to biotinylation assay and the biotinylated (surface) and non-biotinylated (lysate) protein fractions were blotted with anti-Cdo, anti-Kir2.1 or anti-Myogenin antibodies. Myogenin was used as a negative control for the surface protein fraction. The protein level was quantified by using Image J. The level of each biotinylated fraction was normalized to that of the corresponding non-biotinylated protein fraction. The relative surface fraction level of each protein at DM0 was set to 1.0. (C) Confocal immunofluorescence detection of Cdo-GFP (green) and Kir2.1 (red) in C2C12 cells, which were differentiated for 8 hours after switching into DM. Cell nuclei were visualized by DAPI (blue). The phase contrast image is shown, and the scale bar indicates 20μm. (D) The control or Cdo-depleted C2C12 cells were induced to differentiate for indicated time, and subjected to biotinylation assay. The biotinylated (surface) and non-biotinylated (lysate) protein fractions were blotted with an anti-Kir2.1 antibody. Cadherin was used as a loading control. The relative amount of each biotinylated protein was normalized to the level of Cadherin. The relative surface Kir2.1 level of the control at DM0 was set to 1.0.

Because the decreased Kir2.1 activities in Cdo-deficient myoblasts occurred without the decline of Kir2.1 levels, we hypothesized that Cdo might regulate the activity of Kir2.1 via other regulatory mechanisms such as surface translocation or open probabilities [19,20]. First of all, we assessed whether Cdo could interact with Kir2.1 in C2C12 myoblasts. As shown in Fig 3A, Cdo and Kir2.1 formed a complex in C2C12 cells at DM0 and DM1, and then decreased as the differentiation proceeded. Since both proteins function at the plasma membrane, we tested whether the surface localization of these proteins were altered during myoblast differentiation by performing the surface biotinylation. The surface resident Cdo and Kir2.1 proteins were found in C2C12 cells at DM0 and the membrane residences were further ascended at DM1 (Fig 3B), when Kir2.1 is known to be activated to induce membrane hyperpolarization which is important to induce myogenin gene expressions [17,18]. Then, in order to confirm the translocation of Cdo and Kir2.1 proteins during the early myogenic differentiation, we performed immunocytochemistry (Fig 3C). To visualize the expression of Cdo more clearly, C2C12 cells were transfected with GFP-tagged Cdo expressing vector, and then induced to differentiate for 8 hours after switching into DM. The results revealed that both proteins were notably observed in the plasma membrane, where cell-cell interaction occurred, and furthermore co-localized together. Next we have assessed the effect of Cdo depletion on the localization of Kir2.1 proteins at the plasma membrane. C2C12 cells were stably transfected with the control (pSuper) or Cdo shRNA expression vectors and were subjected to the surface biotinylation at the initial stages of differentiation. After 4 hours of differentiation induction, C2C12/pSuper cells exhibited strong but transient induction of total and membrane-resident Kir2.1 protein. The level of membrane-resident Kir2.1 protein was increased after 4 hours and remained high until 8 hours of DM switch while, the Kir2.1 total protein level was decreased to the basal level. Interestingly, in C2C12/Cdo shRNA cells, slightly higher levels of Kir2.1 proteins at the cell surface were found at DM0. However, these were unchanged after 4 hours of DM in these cells and further decreased after 8 hours of differentiation, while total protein levels slightly increased (Fig 3D). These data suggest that Cdo depletion perturbed the induction and membrane translocation of Kir2.1 upon induction of differentiation contributing to decreased Kir2.1 activation.

**Fig 4:**
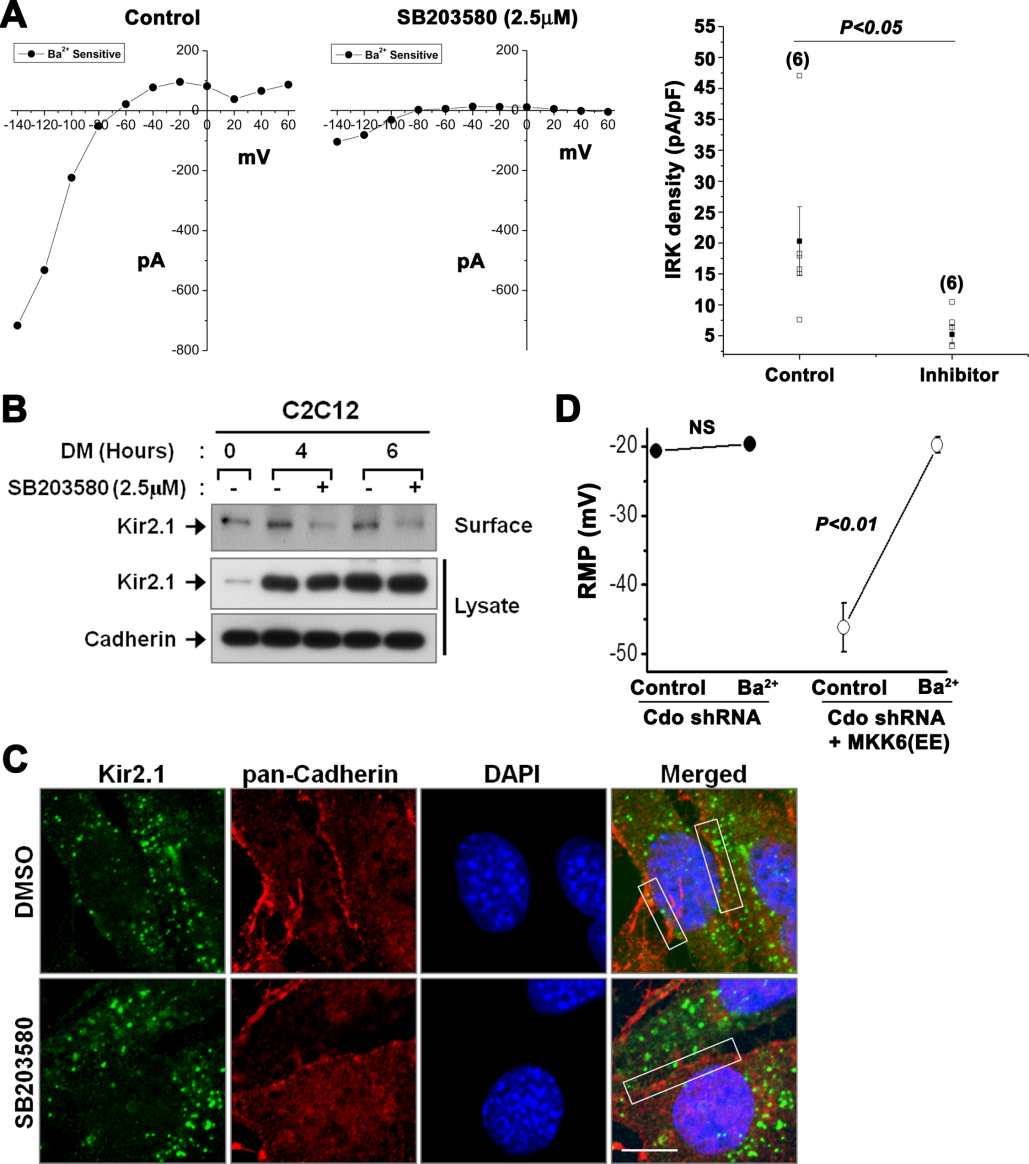
p38MAPK signaling was required for the translocation of Kir2.1 to the plasma membrane. (A) Current-voltage relationships of inwardly rectifying K^+^ (IRK) current of C2C12 cells in the absence (left) or the presence (middle) of SB203580 (2.5 μM). The current density of IRK at -140 mV before or after SB203580 is shown on the right. (B) DMSO (0.25%)- or SB203580-treated C2C12 cells were induced to differentiate for indicated time, and subjected to biotinylation assay. The biotinylated (surface) and non-biotinylated (lysate) protein fractions were blotted with an anti-Kir2.1 antibody. Cadherin was used as a loading control. (C) Confocal immunofluorescence detection of Kir2.1 (green) in DMSO-, or SB203580-treated C2C12 cells after differentiation induction for 6 hours. Cell membrane and nuclei were visualized by pan-Cadherin (red) and DAPI (blue), respectively. The regions of plasma membrane, where cell-cell contact occurs, are indicated with rectangles. The scale bar denotes 10μm. (D) Changes in resting membrane potential (RMP) in Cdo-depleted C2C12 cells with or without MKK6(EE).

p38MAPK signaling pathway has been shown to be a major regulatory mechanism for myoblast differentiation and blocking its activity resulted in impaired myoblast differentiation [35,36,37,38,39]. Furthermore, Cdo-mediated promyogenic function is largely regulated through the activation of p38MAPK and MyoD transcription factor to induce Myogenin expression [7,34]. In our previous studies, we have shown that the decline of myoblast differentiation caused by Cdo deficiency correlates with the reduced p38MAPK activation, and reactivation of p38MAPK by expression of a constitutively active form of an upstream kinase MKK6(EE) restores the differentiation capacity of Cdo deficient myoblasts [7,34,40]. Thus, we asked whether p38MAPK activity was required for the activity of Kir2.1 channels. C2C12 cells were induced to differentiate in the presence of the vehicle DMSO or a p38MAPK inhibitor, SB203580 for 4 hours and subjected to measurement of channel activities. Interestingly, p38MAPK inhibitor treatment inhibited Kir2.1 activities significantly (Fig 4A), suggesting that p38MAPK is required for Kir2.1 activities induced upon differentiation. Next, we asked whether the effect of p38MAPK inhibition on the channel activities was regulated via modulation of the membrane translocation of Kir2.1. Since the experimental procedure of Kir2.1 channel activity recording takes roughly 2 hours, cells were treated with the vehicle DMSO or SB203580 for 4 or 6 hours in DM and subjected to the surface biotinylation (Fig 4B). Upon induction of differentiation, the level of total Kir2.1 protein similarly increased in both control and SB203580-treated cultures. However, SB203580 fully inhibited the increase in membrane-resident Kir2.1 level observed at the early stages of differentiation. To confirm the results, we assessed the expression of Kir2.1 by immunocytochemistry with DMSO-, or SB203580-treated C2C12 cells, which were induced to differentiate for 6 hours (Fig 4C). As expected, even though most of Kir2.1 expressions of the control cells were observed in cytoplasm, quite a number of Kir2.1 proteins were detected at the cell membrane where cell-cell contact, which is a critical event for myogenic differentiation, occurred. However, in SB203580-treated cells, the translocation of Kir2.1 to membrane was largely diminished. These data suggest that p38MAPK regulates the translocation of Kir2.1 to the plasma membrane. Next we tested whether the expression of a constitutive form of the upstream kinase for p38MAPK, MKK6(EE) in Cdo-depleted C2C12 cells can restore Kir2.1 activities in differentiating myoblasts (Fig 4D). Consistent with the low Kir2.1 activities (Fig 1G), the resting membrane potential (RMP) of Cdo-depleted C2C12 cells was low (-20.66±0.88 mV) and it was not changed by Ba^2+^ (-20±1 mV, *n* = 4; NS). As expected, MKK6(EE) induced a hyperpolarization of Cdo-depleted C2C12 cells to -46.16±3.5 mV, which was back to -19.83±1.1 mV (*n* = 6, *P*<0.01) by Ba^2+^, indicating hyperpolarization was due to restoration of Kir2.1 activity. These data suggest that Cdo regulates the activity of Kir2.1 by facilitating cell surface expression via p38MAPK signaling upon myoblast differentiation.

## Discussion

Human myoblasts must be hyperpolarized prior to the induction of differentiation [41], and this hyperpolarization occurs via an increase in Kir2.1 K^+^ channel activity [16]. Hyperpolarization of myoblasts induces a Ca^2+^ influx that is an essential early step of the differentiation process [42]. However, molecular mechanisms underlying Kir2.1 channel activation remain largely unknown. Here, we showed that Kir2.1 activity at the onset of differentiation might depend on Cdo signaling strength. Cdo resides at surface membrane, serving as a multifunctional coreceptor with mechanistically distinct roles in multiple signaling pathways. During differentiation, Cdo links cadherin based cell–cell adhesion to a defined signaling pathway (Cdo/p38MAPK) that directly regulates the differentiation program [6,7,34,40]. Therefore, Kir2.1 activity could be initiated during differentiation by cell-cell adhesion signals through Cdo/p38MAPK complex. A recent study showed the major role of tyrosine phosphatases in regulation of Kir2.1 activity during myoblast differentiation [43]. It proposed that, in human proliferating myoblasts, Kir2.1 channels are kept silent at the plasma membrane by phosphorylation of tyrosine 242, and that these channels are activated in response to extracellular stimuli that induce their dephosphorylation. This dephosphorylation process is mediated by a reduction of growth factor and an increase of phosphatase activity. In accordance with this, we do not exclude a possibility that Cdo signaling pathway may be involved in the modulation of Kir2.1 activities. Our data demonstrated that Kir2.1 activity is regulated by Cdo-mediated signaling pathways during myoblast differentiation. The control of the Kir2.1 channel activity throughout differentiation is thus likely to be dependent on a fine tuning of both Cdo and tyrosine phosphatase levels, one for channel trafficking and the other for gating properties.

The function of ionic channels at the plasma membrane can be mediated by various intra- and extracellular signaling pathways. Members of the inward rectifying K^+^ channel family possess multiple PIP_2_-binding sites that are known to be key regulators of their gating properties [25,26]. Furthermore, Kir2.1 can be modulated by PKA [27], PKC [28,29], and receptor-activated tyrosine kinases [30,31,32,33]. Recently, it has been suggested that kinases and phosphatases can be intimately associated with channels in a single regulatory protein complex that modulates channel activity [44]. However, the regulation of ion channels is not likely to involve just modulation of their synthesis and gating properties. Additionally, their trafficking and localization seem to be also important. Kir2.1 channels have several trafficking motifs such as an ER- [45,46] and Golgi-export motif [47,48]. In fact, mutation in C-terminus of Kir2.1 that blocks Golgi export results in Anderson-Tawil syndrome, a skeletal and cardiac muscle disease with developmental features [48]. In addition, binding sites for anchoring proteins such as filamin A [22], PSD93 [23] or SAP97 [24] are thought to stabilize Kir2.1 channels at the plasma membrane. We showed that p38MAPK positively regulates the trafficking of Kir2.1 from preexisting intracellular pools to the plasma membrane. Indeed, recent studies showed a role of p38MAPK in the trafficking of ion channels such as large-conductance Ca^2+^-activated K^+^ channels (K_Ca_) [49,50]. The effect of p38MAPK on K_Ca_ is caused, in part, by stabilization of a cortical filamentous actin (F-actin) barrier. Given the early assembly of F-actin network during myoblast differentiation [51,52], p38MAPK effect on Kir2.1 channels might be also mediated via modulation of F-actin. It remains to be addressed in future. In summary, Cdo regulates Kir2.1 activities by p38MAPK, specifically through regulation of surface levels of Kir2.1 at the onset of myoblast differentiation. This novel pathway for regulating surface expression of Kir2.1 channels in myoblasts provides insights into the complex functional regulation of Kir2.1 as well as skeletal muscle differentiation.

## Materials and Methods

### Cell culture and transfection

Myoblast C2C12 cells, purchased from ATCC (ATCC^®^ CRL-1772), were cultured as described previously [34]. In order to induce myogenic differentiation, C2C12 cells were cultured in Dulbecco modified Eagle’s medium (DMEM) with 15% fetal bovine serum (FBS) (growth medium, GM). At near confluence, the growth medium was switched to DMEM with 2% horse serum (HS) (differentiation medium, DM) and then, cells were led to myotube formation for 2 or 3 days. The quantification of myotube formation was determined by a transient differentiation assay described earlier [34]. To generate stable C2C12 cells expressing Cdo-GFP, Kir2.1 or Cdo shRNA, cells were cultured and selected in the medium containing puromycin after transfecting with a plasmid and Lipofectamine 2000 (Invitrogen, Carlsbad, CA). The HA-tagged Kir2.1 expression vector was kindly provided by Dr. Penelope Jones. pBabePuro/Cdo-GFP and pSuper/Cdo shRNA were described previously with all necessary controls [53]. Primary myoblasts were isolated from *Cdo*^+/+^ and *Cdo*^−/−^ mice and cultured as explained earlier [15]. Briefly, all mice were aged between 7 and 12 weeks (n = 3, each) and euthanized by CO_2_ asphyxiation. Satellite cells were isolated from hindlimb muscles by treating Collagenase/Dispase (Sigma-Aldrich, St Louis, MO) in PBS for 30min at 37°C. For satellite cell culture, F10 medium with 20% FBS and basic fibroblast growth factor (bFGF) (100ng/ml) was used and exchanged with fresh medium every day to prevent differentiation. To induce differentiation, cells were kept without adding of fresh medium.

All animal studies were reviewed and approved by the International Animal Care and Use Committee (IACUC) of Sungkyunkwan University School of Medicine (SUSM) (Permit Number: 001004). SUSM is an Association for Assessment and Accreditation of Laboratory Animal Care international (AAALAC International) accredited facility and abides by the Institute for Laboratory Animal Research (ILAR) guide.

### Immunocytochemistry and microscopy

MHC expression was visualized by immunocytochemistry as described earlier [34]. For fixation, cells stably transfected with the indicated vector were incubated with 4% paraformaldehyde (PFA) for 20min, and followed by permeabilization with 0.5% Triton X-100 in PBS for 10min. After blocking with 5% calf serum for 1 hour, the cells were probed with anti-MHC antibody (1:500, hybridoma bank) for overnight. Then, incubation with Alexa Fluor^®^ 488 goat anti-mouse antibody (1:500, Invitrogen) was performed for 2 hours at RT. Nikon ECLIPSE TE-2000U microscope and NIS-Elements F software (Nikon) were employed to capture and process the images. The extent of myotube formation was quantified by determining the percentage of nuclei in myotubes. The number of nuclei in MHC-expressing cells was counted, and scored as mononucleate, containing two to four nuclei, containing five to nine, or containing ten or more nuclei.

For confocal imaging, differentiated C2C12 cells were immunoreacted with anti-GFP (1:300) (AbFrontier, South Korea), anti-Kir2.1 (1:200) (Santa Cruz Biotechnology, Santa Cruz, CA), or anti-pan-Cadherin (1:200) (abcam, Cambridge, UK) antibody, and detected using immunofluorescence. Confocal microscopy was performed at Sungkyunkwan University, School of Medicine-Microscopy Shared Resource Facility with Zeiss LSM-510 Meta confocal microscope.

### Surface biotinylation and western blotting analysis

Surface biotinylation was carried out as explained earlier. [54]. In brief, C2C12 myoblasts were cultured for various differentiation times, after which the surface proteins were biotinylated by exposure to NHS-LC-biotin (Thermo) in PBS for 30min at 4°C. After quenching with 100mM glycine, cells were lysed in lysis buffer (10mM Tris-HCl pH7.2, 150mM NaCl, 1% Triton X-100, 1mM EDTA) with proteinase inhibitor (Roche Diagnostics) for 1hr at 4°C. Biotinylated proteins were collected from streptavidine-agarose beads (Pierce), then the samples were analysed by SDS-PAGE and PVDF-membrane transfer.

Western blot analysis was conducted as explained earlier [40]. In brief, cell lysis was performed with the lysis buffer composed of 10 mM Tris-HCl (pH 8.0), 150 mM NaCl, 1 mM EDTA, 1% Triton X-100 and complete protease inhibitor cocktail (Roche Diagnostics, Indianapolis, IN) and then, the prepared samples were separated by SDS-PAGE. The antibodies used were as follows. Anti-Kir2.1 (1:500), anti-Myogenin (1:500), anti-β-tubulin (1:1000) (Santa Cruz Biotechnology, Santa Cruz, CA), anti-pan-Cadherin (1:1000) (Sigma-Aldrich, St Louis, MO), anti-CDO (1:500) (R&D Systems, Minneapolis, MN) and anti-MHC (1:500) (MF20: Developmental Studies Hybridoma Bank, Iowa, IA).

### Electrophysiological recordings and data analysis

Current measurements were made with the whole-cell patch clamp technique. Voltage clamp was performed by using an EPC-10 amplifier (HEKA Instrument, Germany) and filtered at 10 kHz. The patch pipettes (World Precision Instruments, Inc., USA) were made by a Narishige puller (PP-830, Narishige Co, Ltd., Japan). The patch pipettes used had a resistance of 2–3MΩ when filled with the pipette solutions described below. All recordings were carried out at room temperature. The normal external solution was as follows: 143mM NaCl, 5.4 mM KCl, 5 mM HEPES, 0.5mM NaH_2_PO_4_, 11.1mM glucose, 0.5mM MgCl_2_, 1.8mM CaCl_2_ (pH 7.4, adjusted with NaOH). The pipette solution was as follows: 110mM KCl, 1mM MgCl_2_, 5mM K_2_-ATP, 10mM 1,2-bis(2-aminophenoxy) ethane *N*,*N*,*N*_,*N*_-tetraacetic acid (BAPTA), 10mM HEPES (pH 7.4 adjusted with KOH). Currents were analyzed and fitted using Patch master (HEKA Instrument) and Origin 6.1 (Originlab Corp., USA) software. All values are given as mean ± SEM. Current densities (pA/pF) were obtained after normalization to cell surface area calculated by Patch master.

### Statistics

Statistical analyses were performed by using Student’s *t* test. *P*<0.05 was considered statistically significant.
